# Comparative exomics of *Phalaris *cultivars under salt stress

**DOI:** 10.1186/1471-2164-15-S6-S18

**Published:** 2014-10-17

**Authors:** Niina Haiminen, Manfred Klaas, Zeyu Zhou, Filippo Utro, Paul Cormican, Thomas Didion, Christian Sig Jensen, Christopher E Mason, Susanne Barth, Laxmi Parida

**Affiliations:** 1 Computational Biology Center, IBM T. J. Watson Research, Yorktown Heights, NY 10598, USA; 2 Teagasc Crops Environment and Land Use Programme, Oak Park Crops Research Centre, Carlow, Ireland; 3 IBM Research, Carlton, VIC 3053, Australia; 4 Teagasc Animal and Bioscience Research Department, Animal & Grassland Research and Innovation Centre, Grange, Dunsany, Co. Meath, Ireland; 5 DLF-Trifolium, Research Division, Store Heddinge, Denmark; 6 Weill Cornell Medical College, New York, NY 10021, USA

**Keywords:** Differential Expression, RNA-sequencing, Salt stress, Transcriptomics, Phalaris, Reed canary grass, Human brain data, Stable genes, Non-parametric model

## Abstract

**Background:**

Reed canary grass (*Phalaris arundinacea*) is an economically important forage and bioenergy grass of the temperate regions of the world. Despite its economic importance, it is lacking in public genomic data. We explore comparative exomics of the grass cultivars in the context of response to salt exposure. The limited data set poses challenges to the computational pipeline.

**Methods:**

As a prerequisite for the comparative study, we generate the *Phalaris *reference transcriptome sequence, one of the first steps in addressing the issue of paucity of processed genomic data in this species. In addition, the differential expression (DE) and active-but-stable genes for salt stress conditions were analyzed by a novel method that was experimentally verified on human RNA-seq data. For the comparative exomics, we focus on the DE and stable genic regions, with respect to salt stress, of the genome.

**Results and conclusions:**

In our comparative study, we find that phylogeny of the DE and stable genic regions of the *Phalaris *cultivars are distinct. At the same time we find the phylogeny of the entire expressed reference transcriptome matches the phylogeny of only the stable genes. Thus the behavior of the different cultivars is distinguished by the salt stress response. This is also reflected in the genomic distinctions in the DE genic regions. These observations have important implications in the choice of cultivars, and their breeding, for bio-energy fuels. Further, we identified genes that are representative of DE under salt stress and could provide vital clues in our understanding of the stress handling mechanisms in general.

## Background

Reed canary grass (*Phalaris arundinacea*) is an economically important forage and bioenergy grass of the temperate regions of the world. Its ability to grow on poor soils also makes it an excellent crop for phytoremediation (mitigating effects of pollution using plants). As a future bioenergy crop, it is targeted for growing on marginal or degraded lands to avoid direct competition with food production. Enhanced resilience against abiotic stresses inherent in such environments would greatly increase the crop's range. Soil salinity is a major factor limiting fertility, either from occasional flooding by sea water, or in more arid areas from irrigation. Analysis of transcriptional changes during exposure to salt stress facilitates identifying key elements of the plant's response pathways and choosing favorable genotypes, leading towards breeding more resilient crops.

Despite its economic importance, *Phalaris *is lacking, to date, a reference genome or even any publicly available genomic data. We undertake the task of a comparative exomic study of different cultivars of *Phalaris *in the context of their response to varying degrees of salt stress. During increasing salt stress, the cultivars were categorized as largely unaffected or severely damaged, depending on their appearance. Two unaffected (positive salt response) and two damaged (negative salt response) cultivars were chosen for RNA sequencing (before and during salt stress). Here there is a requirement to compare meaningfully *apples with oranges*, as opposed to the rather straightforward *apples to apples *paradigm due to juxtaposition of different cultivars under differing conditions. Then the computational method has the burden of having both uncompromised performance as well as resilience to imperfect and incomplete data, i.e., compensate for scant data due to both absence of reference genomes and absence of multitude of replicates in data. Hence the choice of a non-parametric method to detect differentially expressed (DE) transcripts. At the core of such methods is the use of mapping functions: we identify the essential characteristics of such functions and present RoDEO, which uses one such mapping and not only detects DE but also stable genes. Finally, the algorithm is vetted by experimental verification of the results. The non-parametric framework helps in adapting the method across platforms and across species, without any need for re-calibration.

## Results and discussion

### RoDEO evaluation on real human data

While simulation studies are usually effective and serve a useful purpose in evaluating the efficacy of algorithms, we found that in the context of DE detection, the RNA-seq simulators themselves are compelled to use some parametric model for transcript abundances. Also, on most public DE data sets, the gold standard is usually the result of some computational method. To the best of our knowledge, there is no public dataset for stable genes. To avoid any bias we used real human data, where the DE genes were experimentally validated by qRT-PCR. These RNA samples are well characterized and have been used in several benchmarking studies and DE method comparisons [1-3]. We evaluated the performance of RoDEO against parametric DE detectors that assume a negative binomial count distribution model (baySeq, edgeR, sSeq) and demonstrate that it outperforms the others on several scenarios (details in the Methods section). Moreover, RoDEO's parameter-free framework is very suitable for the diverse and challenging data sets of *Phalaris*.

### *Phalaris *reference transcriptome construction

Total RNA was extracted from *Phalaris arundinacea cv Venture *from five different tissues: leaf and stem of mature (40d) plants under drought stress, stem of mature plant under waterlogging stress, shoot and roots of young (9d old) plants. The samples were pooled, converted into a normalized sequencing library, and sequenced on the Roche 454 FLX platform. In total 494, 477 reads with mean length 389bp were assembled into 18, 682 transcripts with the GS De Novo Assembler software from Roche (which has been designed for the analysis of 454 read data), using the default parameters for cDNA analysis and the adapter-trimmed raw data as input. The sequence data are available at the Sequence Read Archive at NCBI, accession number SRP045256.

**Figure 1:**
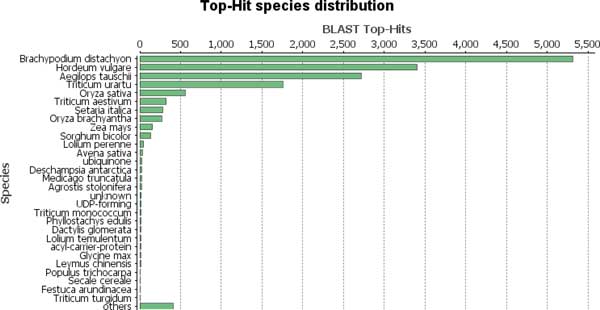
**Top Blast2Go hits' species distribution**. Hits shown for 18, 682 *Phalaris *transcripts. The most matches are to *Brachypodium distachyon*, followed by other related annotated plant species.

All assembled transcriptome contigs were searched against the non-redundant (nr) database at the National Center for Biotechnology Information (NCBI) using BLAST [4]. Gene Ontology annotations for cellular component, biological and molecular processes were predicted for the nr derived BLAST hits using Blast2GO [5]. In addition, InterPro [6] scans were performed to predict protein domain structure of the assembled contigs and Kyoto Encyclopedia of Genes and Genomes (KEGG) [7] pathway assignments were estimated based on enzyme commission (EC) numbers. The distribution of top BLAST hits among annotated plant species are shown in Figure 1. The largest number of sequences match purple false brome (*Brachypodium distachyon*). The species list reflects the known phylogenetic affiliations of *Phalaris*, thus further validating its evolutionary relation to these species. Note that large-scale genomic or transcriptomic datasets have not been previously published on *Phalaris*.

### Salt stress experiment

**Figure 2:**
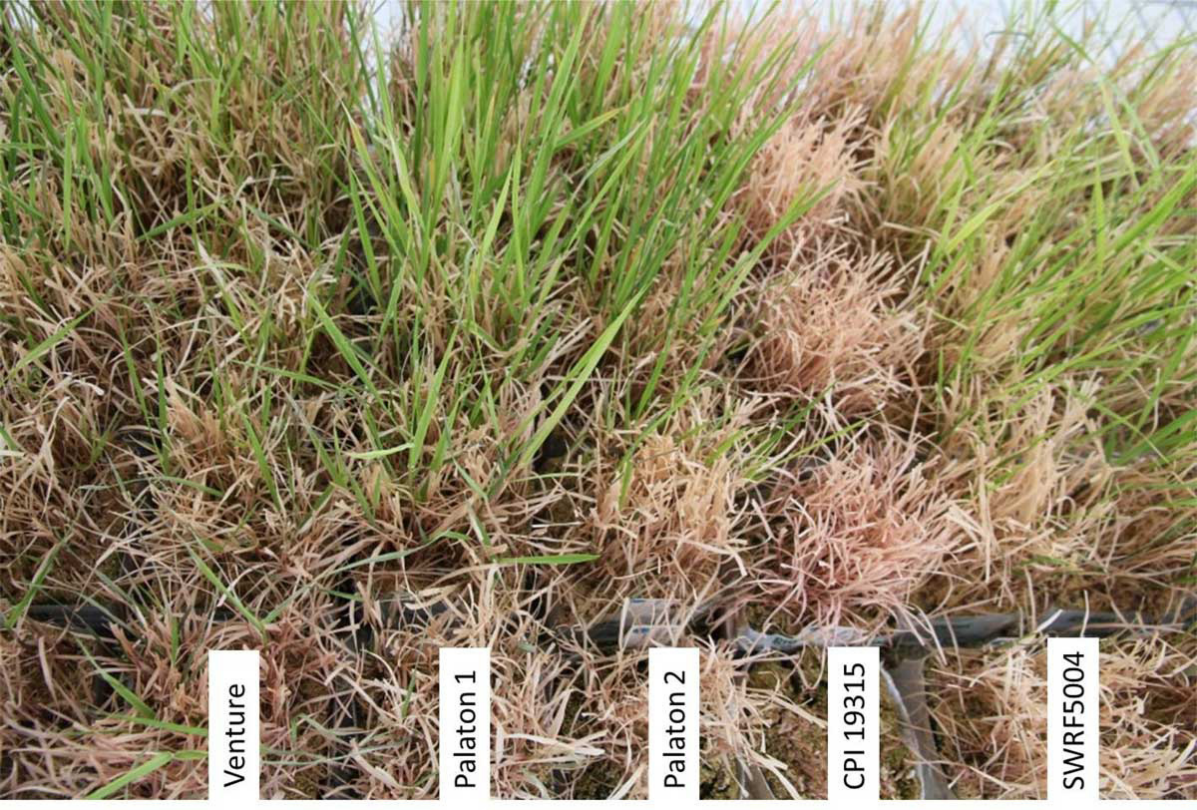
**Images of reed canary grass cultivars**. Cultivars show positive (+) or negative (-) response after 95 days of salt stress. Palaton 2 (+) and CPI 19315 (-) are included in this study. Each label represents five blocks vertically up from the label.

Seeds of three *Phalaris arundinacea *and one *Phalaris aquatica *genotypes were obtained from Lantmännen lantbruk (former Svalöf Weibull seed). SWN RF 9901 is a Norwegian accession from the Nordic Seed bank (NGB7147), SW RF 5008 is a SW breeding line containing European and North American material. Palaton is a OECD listed cultivar in Estonia, Finland, and United States. CPI 19315 is a Moroccan accession of *P. aquatica*, a species closely related to *P. arundinacea *[8]. Two cultivars (CPI 19315, SWN RF 9901) exhibit a negative response to stress, while the other two (Palaton 2, SW RF 5008) tolerate salt stress having a positive response. Figure 2 illustrates some of their phenotypes during salt stress. CPI and Palaton data were collected 55 days after starting the salt treatment, thus called *early *stress, while SWN and SW data were collected after 100 days salt stress, called *late *stress. At sampling time the plants' response to salt of was termed either positive, if the plants looked largely unaffected, or negative, if the plants were severely damaged.

RNA-seq libraries during salt stress and control libraries before salt stress were constructed using the Illumina TruSeq protocol and chemistry (version 2). Libraries were sequenced on Illumina HiSeq2000. The sequence data are available at the Sequence Read Archive at NCBI, accession number SRP045256. The reads were mapped with bwa [9] to the reference transcriptome. In each experiment, 44 - 62 million reads mapped to the reference, with fewer than 10% transcripts having no reads mapping to them.

See Additional File 1 for more details on the salt stress experiment and RNA sequencing.

### Differential expression

We applied our differential expression method RoDEO on the *Phalaris *salt stress read counts per transcript, with parameters *P *= 15, *I *= 100 iterations, *R *equaling the number of mapped reads per experiment, and DE threshold dist(·, ·) = 1.0 and mode distance ≥ 5 (see Methods for details). Stable transcripts were identified by pairwise comparison of the re-sampled iterations, and taking the intersection of the stable sets from all the iterations (see Additional file 1 for details and results).

**Figure 3:**
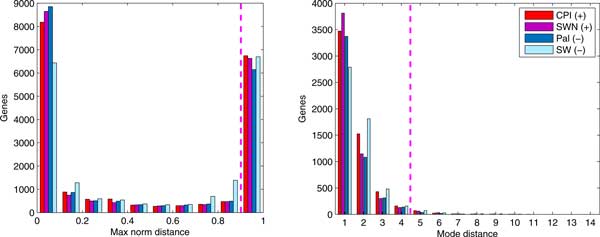
**RoDEO transcripts' differences between stress and control**. Results shown for the salt stress experiments (using max. norm distance). Right: For those about 5, 000 transcripts per cultivar with max. norm distance 1.0, the mode distance distribution is shown. Threshold ≥ 5 is used to identify the most differentially expressed transcripts. Here each color corresponds to the comparison between one cultivar's stress and control samples.

The general differential expression landscape for each cultivar is shown in Figure 3. Thousands of genes have the maximum norm distance 1.0 between their character functions in the control and stress sample, and those transcripts are further ranked by their mode distance to refine the DE candidates set to the most interesting transcripts, i.e., those with mode distance ≥ 5.

**Figure 4:**
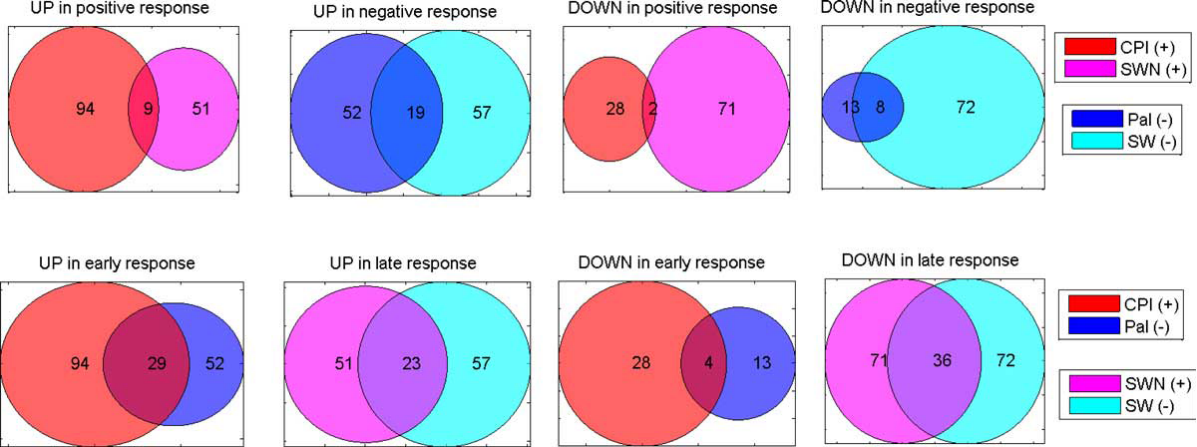
**RoDEO DE transcripts in the salt stress experiments**. Results shown for positive/negative stress response (top), and early/late stress (bottom). The robust transcripts are the intersections of DE transcripts per cultivar. There are two overlaps between the robust up-regulated transcripts in positive/negative and early/late stress responses. Each color represents a cultivar with the number of DE transcripts up or down in that cultivar's stress sample compared to its control sample. The numbers represent the transcripts in each set (intersection included).

We call *robust *positive up-regulated transcripts those that are up-regulated in both positive response cultivars (similarly for down-regulation, and for negative response). These transcripts are more likely to be truly differentially expressed, since they are found DE in both cultivars having the same stress response. Figure 4 shows the overview of the number of transcripts that are up- or down-regulated in each cultivar.

We find 8 robust down-regulated and 19 robust up-regulated transcripts in negative response cultivars. In positive response cultivars, 2 out of 9 robust up-regulated transcripts are shared with the negative response. In addition, there are 2 down-regulated transcripts for positive response. Thus we have in total 10 down-regulated and 26 up-regulated response-specific robust DE transcripts. Annotations for these transcripts are included in Additional file 2.

The annotations for these robust response-specific DE transcripts show several proteins up-regulated under positive response which are widely associated with plant stress proteins, such as *sulphur-rich thionin like protein, thiol protease, polyamine oxidase, universal stress protein a like protein*, and *maize proteinase inhibitor*. Interestingly, these proteins do not show up in the plants with negative stress response, indicating that absence of these proteins could be correlated with a poor response to stress. Instead, enzymes from various catabolic processes are up-regulated which are not specifically active in stress response, such as amino acid or carbohydrate metabolism.

In addition, we find robust DE transcripts for early and late stress response. There are 4 and 36 down-regulated transcripts in early and late stress, respectively. Out of 29 for early and 23 for late up-regulated transcripts, 2 are shared between early and late. Thus we have in total 40 down-regulated and 50 up-regulated time-specific robust DE transcripts. Annotations for these transcripts are included in Additional file 2.

The functional distinction between early and late DE up-regulation is less clear than between positive and negative response. In both early and late categories several proteins associated with abiotic stress response appear, such as *cbl-interacting protein kinase 1-like protein, metallothionein-like protein type 2, delta-1-pyrroline-5-carboxylate synthase-like *(early up-regulated), and e.g. *cysteine proteinase inhibitor, sulphur-rich thionin like protein, thiol protease *(late up-regulated). There is little overlap between the two categories, indicating that the plant response changes between early and late (and more severe) phase of salt stress. The late phase of salt stress reveals proteins that are more similar to the DE proteins detected in the positive response plants that were better able to cope with salt stress.

Two transcripts are up-regulated in all cultivars' stress vs. control samples. One of them has a close match (89% similarity) to Cytochrome P450 71C4 protein in barley (the other has a weaker match to a hypothetical *Aegilopsis tauschii *grass protein). Some other members of the Cytochrome P450 gene family have previously been studied w.r.t. salt stress in Arabidopsis [10].

### Cultivar comparative exomics

We performed a global comparative exomics analysis of the *Phalaris *cultivars based on the variations observed in the RNA-seq reads in the stress experiment. Additionally, we looked for SNP variations across cultivars in the candidate transcripts from the DE analysis. Possibly due to the lack of a reference genome, including non-coding regions and promoters, and because we are limited by the sequenced mRNA information, we did not observe any DE loci where both cultivars with positive response would have allele differences from both cultivars with negative response. Globally, we identified a handful of transcripts with such loci, but they did not exhibit differential expression.

**Figure 5:**
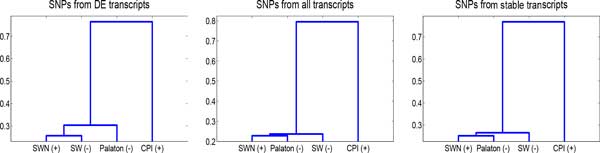
**Phylogenetic trees for the Phalaris cultivars**. The trees are calculated from pairwise distances between cultivars. The trees are built on Hamming distances between the SNPs in Left: DE transcripts, Middle: All transcripts, Right: Stable transcripts.

We computed pairwise distances between the cultivars, based on 200k loci where at least one cultivar's RNA-seq reads differed from the reference transcriptome, and there were at least 100 reads covering the SNP. The dendograms of the pairwise distances (based on Hamming distances of the alleles per loci, and average linkage) are shown in Figure 5. The phylogenetic trees from using all transcripts, DE transcripts, or stable transcripts tell a similar story. Identifying *Phalaris *stable genes is discussed in Additional file 1. CPI (*P. aquatica*), originating from a distant geographical location, is also genetically the most distant from the three North European *P. arundinacea *genotypes. The tree constructed from the DE transcripts differs slightly from the other trees, differentiating Palaton from SWN and SW. It is encouraging that these inferences can be made based on only a partial reference transcriptome and RNA-seq reads from the cultivars.

## Methods

High-throughput RNA-sequencing technologies have taken over the field of gene expression level estimation, previously dominated by microarray technologies. Some of the favorable characteristics of using high-throughput RNA-seq data is that there is no limit on the number of genes surveyed nor do the genes have to be pre-selected. Thus these technologies provide a wider dynamic range and also enable the possibility to discovering new sequence variants and transcripts. Since mRNA sequencing technology is not particularly target specific, up to tens of thousands of genes are "active" at different levels. While this is a fertile ground for functional discoveries, possibly of multiple pathways simultaneously, it is challenged by the onerous task of teasing apart thousands of genes based on their activity (expression) levels across assays. This requires the intervention of reliable computational techniques to make accurate and meaningful inferences from the massive data generated by the experiments. Also, the data is discrete i.e., in terms of read counts or number of read fragments rather than an intensity measurement in a continuous domain. As in all the other computational methods, the following assumptions are made:

1 Most of the genes are stable.

2 The DE genes are globally unbiased, i.e., some are over-expressed while some are under-expressed.

Let *x_g _*be the observed abundance of gene *g*. This is the number of tags (reads) that map to gene *g *in the experiment. The length of the gene is absorbed in this value. Let *µ_g _*be the true abundance. *M_k _*is the library size and *S_k _*represents the total RNA output of a sample. *S_k _*is usually not available but relative RNA production fold change of two samples *S_k_*/*S_k_*′ may be [11].

### The mathematical framework

**Overview**. The primary objective is to compare the expression level of genes in high-throughput sequencing (HTS) data between a pair of biological samples. Due to the un-targeted nature of HTS, it is only natural to assume that the observed expression values of the genes are not independent. Second, the technologies produce data in terms of integer *read counts*. This is in contrast to earlier technologies which produced real-valued intensity measures. Also the total number of genes being simultaneously assayed earlier were determined by the technology while in the sequencing approaches, these read counts are a result of the application of mapping software pipeline, at whose core reside some sophisticated (parametric) string matching algorithms. Such a setting of read count computation is not naturally conducive to normalization processes. Hence the read count of a gene *g *is simply divided by the total number of reads (in millions) in the assay and by the length of the gene *g *(in kilobases) to yield a quantity called the RPKM value of *g*. Instead of directly working with the expression value of *g*, we define a character function $\phi_{g}$ for each gene *g*. The two most desirable properties of this function are (1) $\phi_{g}$ depend on the expression values of all the other genes in the assay and (2) $\phi_{g}$ are scale invariant. We also define another family of distributions, built on these functions (called *ψ*), to study the overall behavior of the changes captured by the two transcriptomes.

### Notation

Let *G *= {*g*_1_, *g*_2_, ..., *g_L_*} be a set of *L *genes. Then let an *L*-tuple of random variables

(1) $$T=\left(X_{g_{1}},X_{g_{2}},\ldots ,X_{g_{L}}\right)={\left(X_{g}\right)}_{g\in G}$$

represent a biological sample with *L *genes *G. X_g _*is a random variable representing the observed expression or abundance of gene *g*. In this model-less framework, we make no assumptions about the distribution of each of the components *X_g_*. Next, consider the *L*-tuple of random variables

(2) $$\text{Φ}\left(T\right)=\left(\phi_{g_{1}}\left(T\right),\phi_{g_{2}}\left(T\right),\;\ldots ,\phi_{g_{L}}\left(T\right)\right)={\left(\phi_{g}\left(T\right)\right)}_{g\in G},$$

whose components, $\phi_{g}$, termed character functions, are defined below. In particular, these functions are scale invariant.

Definition 1 (character functions)

For a fixed P > 1, we call a surjective map

(3) $$\phi ={\left(\phi_{g}\right)}_{g\in G}:{\mathbb{R}}_{\geq 0}^{G}\to \left\{1,2,\ldots ,P\right\},$$

*a character function if there exists a *${\left(\delta_{p}\right)}_{1\leq p\leq P}\in {\mathbb{R}}_{\geq 0}^{P}$, *satisfying the following conditions:*

*1 if *$\phi_{g}\left(t\right)=\phi_{g^{\prime }}\left(t\right)=p$*then |t_g _− t_g′ _| ≤ δ_p_, and*

*2 if *$\phi_{g}\left(t\right)<\phi_{g^{\prime }}\left(t\right)$, *then t_g _< t_g′ _*,

*for all g, g′ ∈ G, and *${\left(t_{g}\right)}_{g\in G}\in {\mathbb{R}}_{\geq 0}^{G}$.

Notice that such a character function satisfies the following additional properties.

1 $\phi_{g}$ is scale-invariant: $\phi_{g}\left(ct\right)=\phi_{g}\left(t\right)$ for all *c *> 0 and $t\in {\mathbb{R}}_{\geq 0}^{G}$.

This follows from the first condition of the definition of $\phi_{g}$.

2 For all triplets, *g, g′, g″*, if *t_g _*<*t_g′ _*<*t_g″ _*with $\phi_{g}\left(t\right)=\phi_{g^{\prime \prime }}\left(t\right)$ then $\phi_{g}\left(t\right)=\phi_{g^{\prime }}\left(t\right)=\phi_{g^{\prime \prime }}\left(t\right)$.

This property follows from the second condition of the definition. In fact this property leads to the algorithms for actually computing the scale-invariant maps.

For two samples *a *and *b *defined on the same gene set *G*, let a pair of *L*-tuples of random variables, $T^{a}$ and $T^{b}$, represent the two samples. Let the corresponding scale-invariant character maps be $\text{Φ}\left(T^{a}\right)={\text{Φ}}^{a}$ and $\text{Φ}\left(T^{b}\right)={\text{Φ}}^{b}$. Recall from Eqn (3) that the image of  ϕ is {1, 2, .., *p*, .., *P*}. Next, consider the *P*-tuple of random variables

(4) $$\Psi \left({\text{Φ}}^{b}|{\text{Φ}}^{a}\right)=\left(\psi_{1}\left({\text{Φ}}^{b}|{\text{Φ}}^{a}\right),\psi_{2}\left({\text{Φ}}^{b}|{\text{Φ}}^{a}\right),\ldots ,\psi_{p}\left({\text{Φ}}^{b}|{\text{Φ}}^{a}\right),\ldots ,\psi_{P}\left({\text{Φ}}^{b}|{\text{Φ}}^{a}\right)\right),$$

whose components $\psi_{p}\left({\text{Φ}}^{b}|{\text{Φ}}^{a}\right)$, termed *dispersion* functions, are defined below.

Definition 2 (dispersion function)

For samples a and b (with possibly a = b), $\psi_{p}\left({\text{Φ}}^{b}|{\text{Φ}}^{a}\right)$ is defined by the following distribution:

(5) $$Pr\left(\psi_{p}\left({\text{Φ}}^{b}|{\text{Φ}}^{a}\right)=q\right)=Pr\left(\underset{g\in G}{\text{V}}\left(\left(\phi_{g}^{a}\left(T^{a}\right)=p\right)\text{Λ}\left(\phi_{g}^{b}\left(T^{b}\right)=q\right)\right)\right),$$

where *q *∈ {1, 2, .., *P*}.

Informally speaking, $\Psi_{p}\left({\text{Φ}}^{b}|{\text{Φ}}^{a}\right)$ is the dispersion of image *p *in sample *b *with respect to that of *a*. Also, $\Psi_{p}\left({\text{Φ}}^{a}|{\text{Φ}}^{a}\right)$ specifies the variance of image *p *in sample *a*. Based on Eqn (5), for each *p*, probability measures of over-expression (*O*) and under-expression (*U*) are defined as follows:

$$\begin{array}{c} {\text{O}}_{p}\left({\text{Φ}}^{b}|{\text{Φ}}^{a}\right)=Pr\left(\psi_{p}\left({\text{Φ}}^{b}|{\text{Φ}}^{a}\right)\geq p+1\right),\\ {\text{U}}_{p}\left({\text{Φ}}^{b}|{\text{Φ}}^{a}\right)=Pr\left(\psi_{p}\left({\text{Φ}}^{b}|{\text{Φ}}^{a}\right)\leq p-1\right), \end{array}$$

Along similar lines, the dispersion within *a*, for each *p*, is

(6) $${\tilde{\text{O}}}_{p}\left({\text{Φ}}^{a}\right)=Pr\left(\psi_{p}\left({\text{Φ}}^{a}|{\text{Φ}}^{a}\right)\geq p+1\right),$$

(7) $${\tilde{\text{U}}}_{p}\left({\text{Φ}}^{a}\right)=Pr\left(\psi_{p}\left({\text{Φ}}^{a}|{\text{Φ}}^{a}\right)\leq p-1\right),$$

Thus a natural measure of neutrality, for each *p*, is

(8) $${\tilde{\text{N}}}_{p}\left({\text{Φ}}^{a}\right)=1-\left({\tilde{\text{O}}}_{p}\left({\text{Φ}}^{a}\right)+{\tilde{\text{U}}}_{p}\left({\text{Φ}}^{a}\right)\right).$$

This intrinsic measure can be used to evaluate the accuracy of the estimation of distributions of random variable ${\text{Φ}}^{a}$, based on single or multiple replicates. In practice, we have used ${\tilde{\text{N}}}_{p}\left({\text{Φ}}^{a}\right)$ to evaluate the character functions  ϕ (and thus estimate parameter *P *as well).

### Estimating distributions of random variables Φ(T) and Ψ(Φ)

Critical to the computations of  and  functions, is the estimation of the distribution of the random variables *X_g _*of Eqn (1). Our model is influenced by the observation that the total number of genes (*L*) in the RNA-Seq experiment is based on the biological sample and not the technology and that this also renders the observed abundances of each gene to be not quite independent of each other. This is reflected in the details of the computations discussed here.

Let $c=\left(c_{g_{1}},c_{g_{2}},\ldots ,c_{g_{L}}\right)$ be the observed abundances in the experiment. Without loss of generality, let *c_g _*be the number of reads (read-counts) of gene *g*, and Σ*_g∈G_c_g _*= *M*. To estimate the distribution we use a Bernoulli process with parameters *c_g_*/*M*, for *g *∈ *G*, and *M*. This is a natural extension of the method for the single replicate. Let ${\left(c_{g}^{1}\right)}_{g\in G},{\left(c_{g}^{2}\right)}_{g\in G},\ldots ,{\left(c_{g}^{K}\right)}_{g\in G}$ be the observed abundances in the *K *experiments under identical conditions. As before, let $c_{g}^{k}$ be the number of reads (read-counts) of gene *g *in the *k*th replicate, for 1 ≤ *k *≤ *K*. Let $M^{k}=\sum_{g}c_{g}^{k}$. We use *K *Bernoulli processes each with parameters $c_{g}^{k}/M^{k}$, for *g *∈ *G*, and *M^k^*, as in the single replicate case. Let $c_{g}=\sum_{k=1}^{K}c_{g}^{k}$ and let $M=\sum_{g}c_{g}$. We estimate the probability, *p_g_*, of gene *g *to be *c_g_*/*M*.

Thus, modeling the experiment, with *K *≥ 1 replicates, as a Bernoulli process, $T={\left(X_{g}\right)}_{g\in G}$ of Eqn (1) follows a multinomial distribution with parameters *M *and (*p_g_*)*_g∈G_*. A closed form of the distribution of $\phi_{g}$ or $\psi_{g}$ is not straightforward to estimate and moreover, the argument to $\phi_{g}$ is an *L*-tuple $t\in {\mathbb{R}}_{\geq 0}^{G}$. Hence we simulate the Bernoulli process in our implementation. Then based on Defn (1), for a fixed *P*, $\phi_{g}$ is evaluated for each *g *using the *L*-tuple, *t_i_*, of trial *i*. Similarly, based on Defn (2), $\psi_{p}$ is evaluated for each *p *using the *L*-tuples of the two experiments. The details are discussed below.

### Simulating the character functions $\phi_{g}$

Simulating the Bernoulli process for one experiment with *n *genes is described below, for some number of iterations *I *(e.g., *I *= 10^3^), and some number of reads *R *(e.g., *R *= 10^6^), using a fixed *P *(e.g., *P *= 20). Consider iteration i:

1 Repeat for each read *r *∈ *R*: Assign the read to gene *g *∈ *G *with probability $p_{g}=\text{log}\;c_{g}/\sum_{i=1}^{n}\text{log}\;c_{i}$.

2 Order genes according to decreasing assigned read count. Assign $\phi_{g}^{i}=1$ for genes *g *with no reads.

3 Apply linear (regression) segmentation with *P *− 1 segments to the cumulative sum of the ordered read counts; assign $\phi_{g}^{i}=P$ for genes *g *in the first segment, *P *− 1 for the second segment, and so forth. Least-squares linear segmentation can be performed in time *O*(*n*^2^*P*), see [12]. In practice when *n *is large one may want to subsample the genes, e.g., only use every 10th gene for the segmentation.

The values $\phi_{g}^{i}$ are stored in each iteration *i *and together make up the distribution $\phi_{g}$. The values of the dispersion function *ψ_p _*are computed from $\phi_{g}$. In practice, to compute $Pr(\Psi_{p}({\text{Φ}}^{b}|{\text{Φ}}^{a})=q)$, one counts the number of pairs of iterations (*i, i*′) ∈ (1...*I*, 1...*I*) where $\phi_{g}^{a_{i}}=p$ and $\phi_{g}^{b_{i^{\prime }}}=q$, where $\phi_{g}^{a_{i}}$ denotes the bin of gene *g *in iteration *i *in dataset *a*. The probability *Pr *is the frequency of pairs $\left(\phi_{g}^{a_{i}}=p,\phi_{g}^{b_{i^{\prime }}}=q\right)$ among all the pairs $\left(\phi_{g}^{a_{i}}=p,\phi_{g}^{b_{i^{\prime }}}=p^{\prime }\right)|p^{\prime }\in 1\ldots P$.

**Figure 6:**
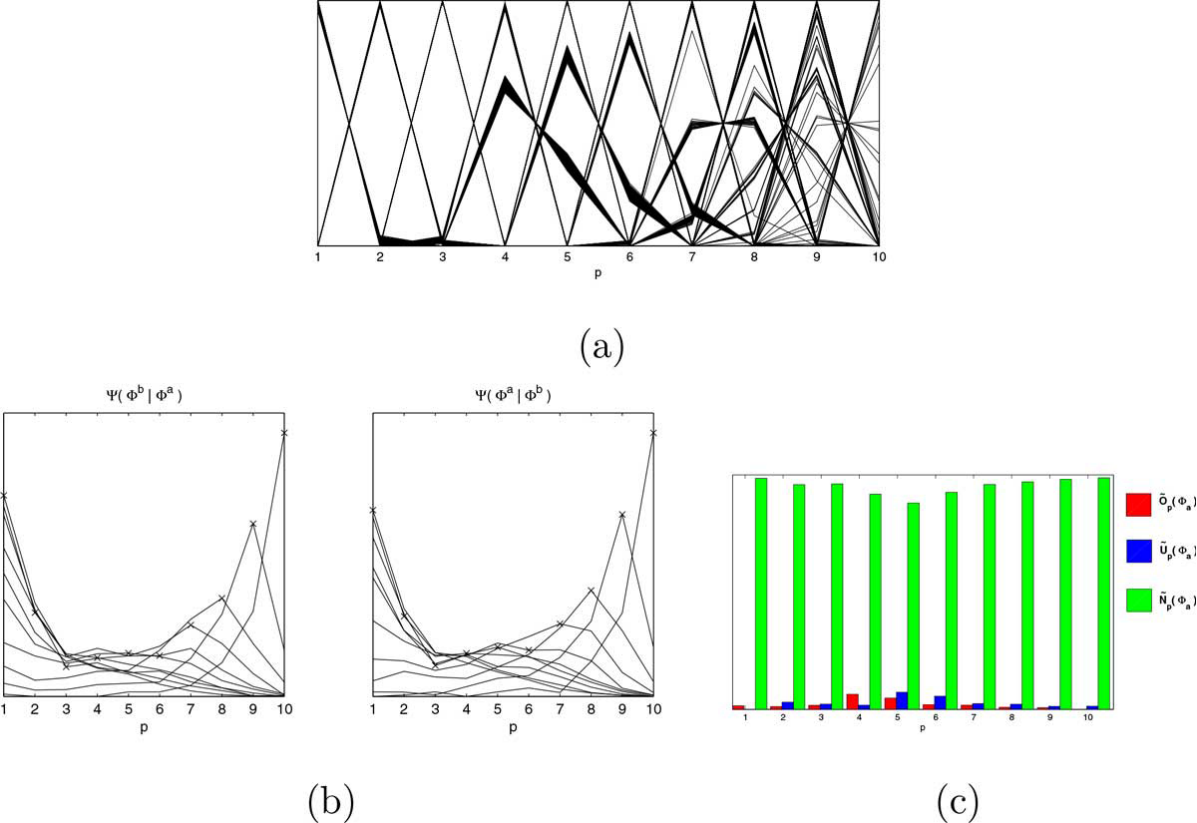
**Illustration of the RoDEO distributions**. (a) $\phi_{g}$ for each gene *g *for 10,000 genes. (b) Distributions $\Psi_{p}\left({\text{Φ}}^{b}|{\text{Φ}}^{a}\right)$ (left), and $\Psi_{p}\left({\text{Φ}}^{a}|{\text{Φ}}^{b}\right)$ for each *p *(Eqn 5). (c) Intrinsic measures of dispersion within dataset *a*, for each *p *(Eqns 6-8).

*An example *We compute the different distributions for a dataset with 10,000 genes and their (observed) abundances *c*. Figure 6 (a) shows $\phi_{g}$ for all the genes *g *(as defined in Eqn 3) and Figure 6 (b) shows the $\Psi_{p}({\text{Φ}}^{b}|{\text{Φ}}^{a})$ and $\Psi_{p}({\text{Φ}}^{a}|{\text{Φ}}^{b})$, for each *p *(according to Eqn 5), while (c) summarizes the Ψ distributions as ${\tilde{\text{O}}}_{p}\left(\text{Φ}\right)$, ${\tilde{\text{U}}}_{p}\left(\text{Φ}\right)$, and ${\tilde{\text{N}}}_{p}\left(\text{Φ}\right)$ (according to Eqns 6-8).

### Comparison across a pair $T^{a}$ and $T^{b}$

Instead of directly studying $T^{a}$ and $T^{b}$, we observe instead the behavior of the scale-invariant maps $\text{Φ}\left(T^{a}\right)$ and $\text{Φ}\left(T^{b}\right)$. We also distinguish two classes of genes: one whose members are *differentially expressed *(DE) the others whose members are *stable *across the two samples *a *and *b *with sufficient confidence (based on a threshold on some measure). The definition of stable genes in this context, and detecting them via the combinatorial problem of longest increasing subsequence is discussed in Additional file 1. Unfortunately, these two which appear complementary in their characteristics do not exactly partition the genes *G *into two because of the need to meet the confidence thresholds.

### Differentially expressed (DE) genes

Character functions $\phi_{g}\left(T^{a}\right)$ and $\phi_{g}\left(T^{b}\right)$ are used to estimate the DE genes. We define a real-valued distance, *dis*(·, ·) ≥ 0, between the two functions as discussed below. For some fixed *δ *> 0, if $dis\left(\phi_{g}\left(T^{a}\right),\;\phi_{g}\left(T^{b}\right)\right)>\delta$, then *g *is estimated to be differentially expressed. It is important to note that the mathematical framework discussed here is agnostic to the underlying models in the diverse read count simulators prevalent in literature.

To define the amount of differential expression between datasets *a *and *b*, we rank each gene according to the differences in their distributions $\phi_{g}\left(T^{a}\right)$ and $\phi_{g}\left(T^{b}\right)$. The ranking process is described below.

The estimated character functions are essentially histograms on *P *ordered bins *x *= 1, ... , *P*, denoted $\phi_{g}\left(T,x\right)$. We adopt *maximum norm *as the DE measurement between treatments, denoting the largest difference between the cumulative distributions for gene *g *in experiments *a, b*:

(9) $$dis{(\phi }_{g}\left(T^{a}\right),\phi_{g}\left(T^{b}\right))=\underset{x}{\text{max}}\left|C\left(\phi_{g}\left(T^{a}\right),x\right)-C\left(\phi_{g}\left(T^{b}\right),x\right)\right|,$$

where *C *denotes the cumulative distribution, $C\left(\phi_{g}\left(T^{a}\right),x\right)=\sum_{i=1}^{x}\phi_{g}\left(T^{a},i\right)$. This is the test statistic in the Kolmogorov-Smirnov test. However, the character functions are discrete, so the K-S test is not applicable. Due to the finite numbers of iterations when estimating the distributions, two genes often have the same maximum norm distance. Then we further compare the genes by the distance between their modes in $\phi_{g}\left(T^{a}\right),\;\phi_{g}\left(T^{b}\right)$. In the case of *K *replicates, $\phi_{g,i}\left(T^{a},x\right),$ is calculated for the *i*th replicate, and we perform DE analysis on the average character functions, $\bar{\phi_{g}}\left(T,x\right)$:

(10) $$\bar{\phi_{g}}\left(T^{a},x\right)=\frac{\sum_{i=1}^{K}\phi_{g,i}\left(T^{a},x\right)}{K}.$$

The genes are ordered by decreasing maximum norm, then by decreasing mode distance, and the genes at the top of the list are the most likely DE candidates.

### Comparison with existing methods

We compare RoDEO against existing methods baySeq [13], edgeR [14], and sSeq [15] for identifying differentially expressed genes. In an effort to avoid biases often associated with generating simulated data using the exact distribution assumed by the methods (negative binomial in this case), we only used real data. Specifically, we used read counts and quantitative expression data from the MAQC human tissue samples, for which DE genes have been experimentally validated by qRT-PCR [1,2]. The aim here is to detect differentially expressed genes between human reference RNA and brain RNA samples.

The methods were run on all 33k genes for which there were reads sequenced with Ion Torrent Proton technology (MAQC PRO dataset), while the DE results were evaluated only on those 17k genes with available qRT-PCR quantification. We used log-fold change cutoff > 1.5 to define DE genes and < 1.0 to define non-DE genes (other genes remained unassigned). Each method was run with default parameters (if any), RoDEO was run with *P *= 20 bins, *I *= 100 iterations, and *R *= 10^7 ^reads. Each compared method provides a list of genes ordered by extent of their DE, and this list is used in the evaluation.

**Figure 7:**
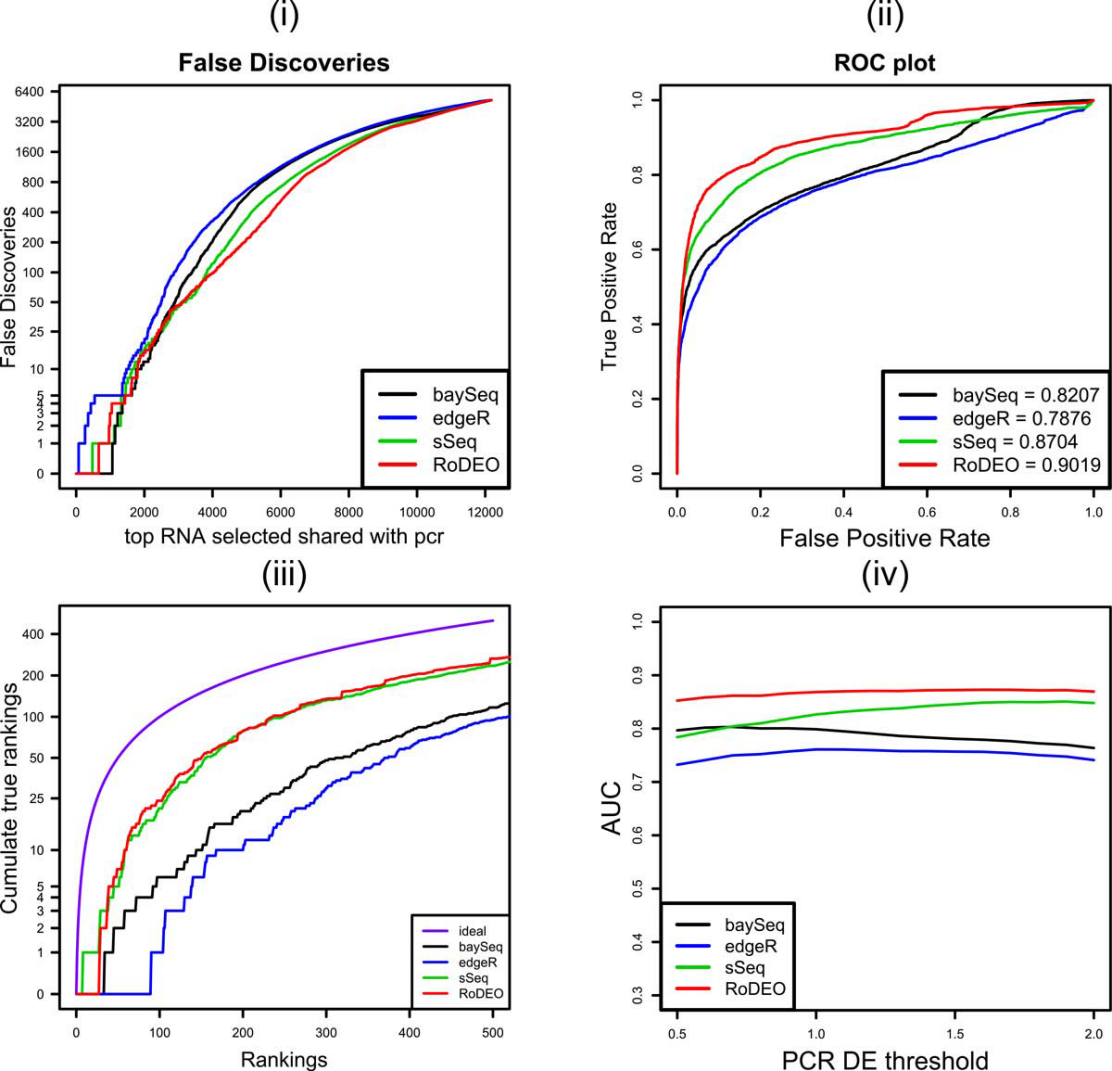
**MAQC PRO results on three replicates**. The four panels show (i) false discoveries in the top DE genes, (ii) false positive vs. true positive rate and area under the curve (AUC) measurement, (iii) number of true DE genes with ranks 1...*x *within top *x *genes by the method, and (iv) AUC when varying the threshold for calling DE genes from the qRT PCR results (threshold 1.5 is used in panels i-iii).

**Figure 8:**
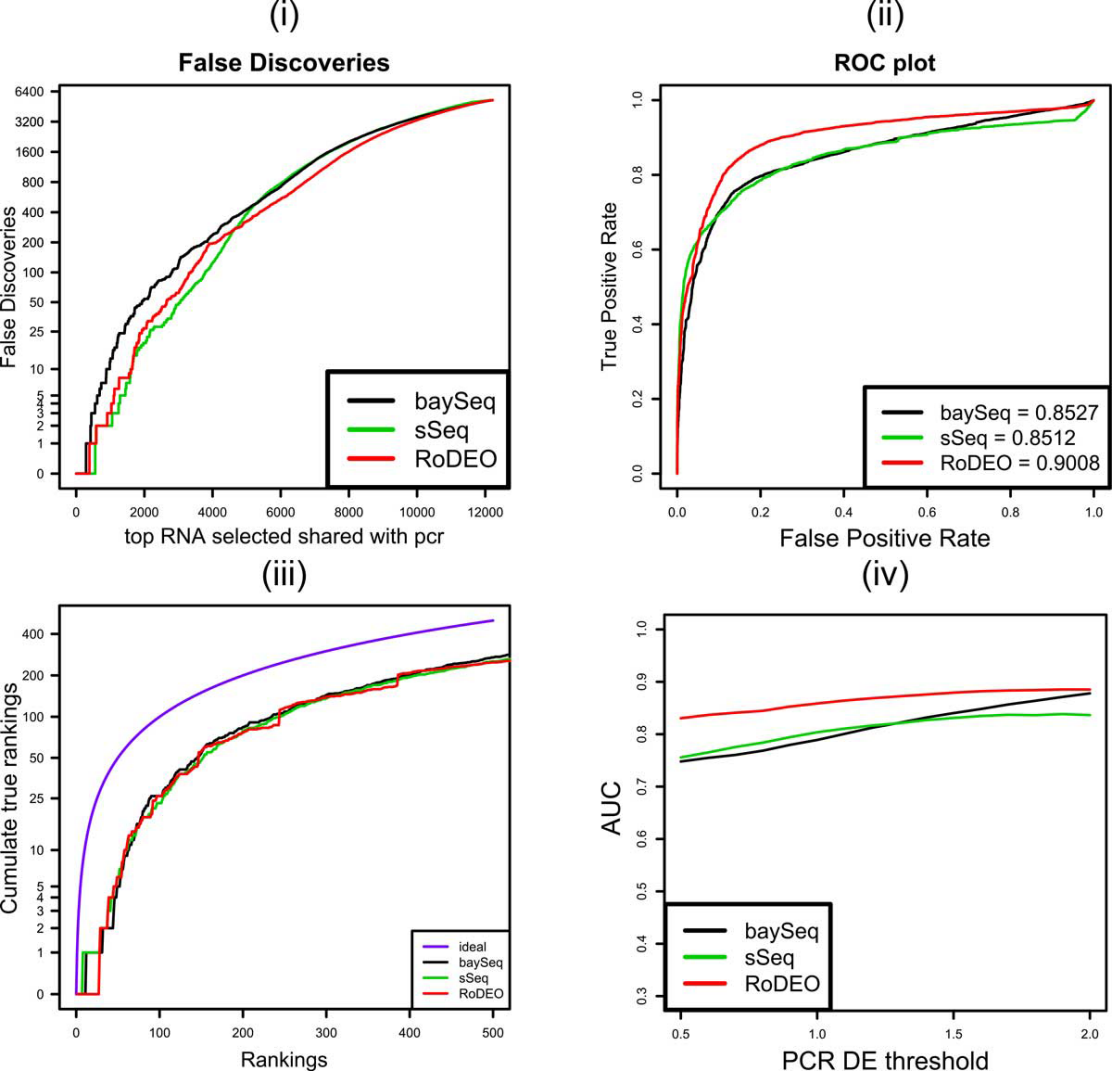
**MAQC PRO results on one replicate**. The four panels show (i) false discoveries in the top DE genes, (ii) false positive vs. true positive rate and area under the curve (AUC) measurement, (iii) number of true DE genes with ranks 1...*x *within top *x *genes by the method, and (iv) AUC when varying the threshold for calling DE genes from the qRT PCR results (threshold 1.5 is used in panels i-iii).

Figure 7 shows the DE methods' performance on the this MAQC PRO data consisting of two treatments, each with three replicates. Results on a single-replicate case using the first replicate only are shown in Figure 8. The four panels illustrate (i) For the number of top DE candidates on the x-axis, how many are false positives, (ii) False positive rate vs. true positive rate, including AUC (Area Under the Curve) measurement, (iii) For the top up to *x *= 500 true DE genes, how many are included in the method's DE list of size *x *(ideally *x *= *y*), (iv) AUC when varying the threshold for calling DE genes from the qRT-PCR results (threshold 1.5 is used in panels i-iii).

RoDEO outperforms all the other DE methods on this dataset, based on the AUC measurement at varying levels of the PCR DE threshold (panel iv), both on the multiple replicate and single replicate cases.

## Conclusion

We present RoDEO, a novel method for detecting differentially expressed and stable genes. RoDEO is vetted by experimental verification on human reference data, where it outperforms existing methods on several scenarios. The non-parametric framework helps in adapting the method across platforms and across species, without any need for re-calibration.

RoDEO is applied to analyze RNA-seq data from reed canary grass salt stress experiments. In the process, the first reference transcriptome is constructed for *Phalaris arundinacea*, an economically important forage and bioenergy grass. Despite the lack of a complete reference genome, including non-coding regions and promoters, and being limited to the sequenced mRNA information, we observe differential expression in four cultivars with distinct genotypes. Furthermore, we study the phylogeny of the DE and stable genic regions, and find the behavior of the different cultivars distinguished by the salt stress response.

## Competing interests

The authors declare that they have no competing interests.

## Authors' contributions

LP and SB designed and coordinated the study. LP, NH, and ZZ developed the differential expression algorithm. NH and FU performed differential expression and comparative exomics analyses on the *Phalaris* data. ZZ carried out the comparison with existing methods. MK prepared the *Phalaris *salt stress data. PC provided *Phalaris *transcriptome annotations. TD and CSJ performed the *Phalaris *salt stress physiological experiment. CEM provided human data, contributing to the comparison with existing methods.

## Supplementary Material

Additional file 1 **Additional methods and results**. Additional method details and results (.pdf). Click here for file

Additional file 2 **DE transcript annotations**. Annotations for the robust differentially expressed transcripts (.xls). Click here for file

## Declarations

Publication costs for this article were funded by the corresponding author's institution.

This article has been published as part of *BMC Genomics *Volume 15 Supplement 6, 2014: Proceedings of the Twelfth Annual Research in Computational Molecular Biology (RECOMB) Satellite Workshop on Comparative Genomics. The full contents of the supplement are available online at http://www.biomedcentral.com/bmcgenomics/supplements/15/S6.
