# Supplementary material for: Comparative exomics of Phalaris cultivars under salt stress
Source: BMC Genomics. 2014 Oct 17;15(Suppl 6):S18. doi: 10.1186/1471-2164-15-S6-S18 (PMC4240679; doi:10.1186/1471-2164-15-S6-S18)
Supplement: Additional file 1 — Additional methods and results. Additional method details and results (.pdf). [file 1471-2164-15-S6-S18-S1.pdf]

Additional file 1

Haiminen *et al.*: Comparative Exomics of *Phalaris* cultivars under salt stress. BMC Genomics 2014.

Phalaris salt stress experiment details

Salt stress experiment *Phalaris* seeds were germinated and grown in five replicates on 10x10x5cm rock wool blocks. The blocks were placed on tables that were intermittently flooded with water of the appropriate salt concentration. After 50d establishment without salt, the plants were subjected to 0.5% NaCl for 30d, then to 1.0% for20d, then to 1.5% for 40d, and afterwards to 2%. Samples for RNA extraction were taken 50d after germination before salt treatment, early into salt treatment (30d 0.5% salt, 20d 1.0% salt, 5d 1.5% salt), and late into salt treatment (a further 36d at 1.5% salt, and 9d 2% salt).

RNA sequencing Sequencing libraries were constructed with the TruSeq RNA sample preparation kit (Illumina) according to the manufacturer's instruction, starting from 1.3 μg total RNA. Four samples were indexed and combined in one Illumina HiSeq sequencing flow cell lane. After sequencing on the Illumina HiSeq 2000 platform, between $50 - 64$M reads per sample were obtained.

Stable genes

Across $\mathcal{T}^a$ and $\mathcal{T}^b$ on the same gene set G, while the abundances of the genes provide a framework for detecting differentially expressed genes, the same is not adequate to tease out the stable or non-changing (nonDE) genes in G. Inspired by the Φ character functions, whose image is a set of positive integers, we use a combinatorial framework to detect the stable genes.

Let $\pi = p_1, p_2, ..., p_l$ be a sequence of positive integers. Further, $len(\pi) = l$ is the length of π and its ith element, p_i, is also written as $\pi(i)$. A sequence $\pi' = p_{i_1}, p_{i_2}, ..., p_{i_{l'}}$ for some $i_1 < i_2 < .. < i_{l'}$ and $l' \leq l$, is called a *subsequence* of π. Further, if $p_{i_1} \leq p_{i_2} \leq .... \leq p_{i_{l'}}$ holds then it is called an *increasing* subsequence.

For example, consider the sequences

$$\begin{aligned}
\pi_1 &= 1\,1\,2\,1\,1\,1\,2\,3\,2\,2\,2\,3 \\
\pi_2 &= 1\,1\,1\,1\,1\,2\,2\,2\,2\,2\,3\,3 \\
\pi_3 &= 1\,1\,1\,1\,1\,1\,2\,3 \\
\pi_4 &= 1\,1\,1\,2\,2\,2\,2
\end{aligned}$$

Here, π_4 is a subsequence of both π_1 and π_2 but not π_3; π_3 is not a subsequence of any of the other three; π_2, π_3 and π_4 are increasing sequences.

Consider ϕ^a and ϕ^b of Eqn(3). Let π^a be an increasing sequence with a one-to-one map

$$\theta : \{1, 2, ..., L\} \rightarrow \{g_1, g_2, ..., g_L\} \tag{11}$$

such that $\pi^a(i) = \phi^a(\theta(i))$. Based on π^a, π^b is defined as follows: for $1 \leq i \leq L$, $\pi^b(i) = \phi^b(\theta(i))$. Notice that, in effect, π^b is a re-labeling of ϕ^b in terms of ϕ^a. Consider a concrete example with at least two possible θ maps, θ_1 and θ_2, and their corresponding sequences π^a and π^b.

$$\begin{array}{lllllll}
\phi^a = 2\ 1\ 1\ 2\ 4\ 3 & i & & 1\ 2\ 3\ 4\ 5\ 6 & i & & 1\ 2\ 3\ 4\ 5\ 6 \\
\phi^b = 1\ 4\ 1\ 3\ 2\ 1 & \theta_1(i) & & 2\ 3\ 1\ 4\ 6\ 5 & \theta_2(i) & & 3\ 2\ 4\ 1\ 6\ 5 \\
 & \pi^a & = & 1\ 1\ 2\ 2\ 3\ 4 & \pi^a & = & 1\ 1\ 2\ 2\ 3\ 4 \\
 & \pi^b & = & 4\ 1\ 1\ 3\ 1\ 2 & \pi^b & = & 1\ 4\ 3\ 1\ 1\ 2
\end{array}$$

Next, a stable set of ϕ^a and ϕ^b is modeled as a *longest increasing subsequence* of π^b. This problem is defined below.

Definition 3 (Longest Increasing Subsequence (LIS) Problem) *Given an integer sequence π, let Π be the set of all subsequences of π. Then π' is a longest increasing subsequence of π if and only if $len(\pi') = \max\{len(\pi'') \mid \pi'' \in \Pi\}$.*

The LIS problem can be solved exactly in polynomial time as described below.

Solving the Longest Increasing Subsequence (LIS) problem

The LIS problem can be solved exactly in time $\mathcal{O}(n \log n)$ using dynamic programming, see e.g. [1]. However, when we want to keep track of all possible alternative

LIS solutions the time complexity becomes $\mathcal{O}(n^2 + N)$, where N is the number of alternative LIS solutions (see the discussion below).

Two distinct subsequences π' and π'' are mutually longest increasing subsequences if $len(\pi') = len(\pi'')$ and the subsequences are increasing. In the worst case, the number N of alternative LIS solutions is exponential, i.e. $(\mathcal{O}(1))^n$, for a sequence of length n. Consider the following example: $\pi = 13246579810121113151 4....n$ Then it can be verified that all the maximal LIS are obtained by picking one entry from the two elements in each of the square brackets: $1[3,2]4[6,5]7[9,8]10[12,11]13[15,14]....n$.

Dynamic programming relies on the fact that the LIS solutions for the prefixes of sequence S can be utilized when computing the LIS for S. The algorithm iteratively finds the LIS ending at position i, $i = 1, 2, ..., n$, where n is the number of elements in sequence S. Sequence S is the re-labeled sequence π^b in the case of stable genes.

Denote the maximum size of a LIS ending at position j as M_j. When processing element S_i, the algorithm checks all the thus far largest $M_j, j < i$, for which $S_i \geq S_j$ and extends their LIS with S_i. There may exist several j with the same size M_j, and they all need to be examined as possible alternative solutions. Consider for example a pathological case $S = 7, 6, 5, 3, 2, 1, 4$. The LIS size for S is 2 and the possible solutions are $(3, 4)$, $(2, 4)$, $(1, 4)$. When considering the last element $S_n = 4$, one needs to find all the positions $j < n$ with the maximal M_j (in the example maximal $M_j = 1$, $j = 1, ..., 6$). The elements S_4, S_5, S_6 need to be linked to S_n as potential prefixes of the LIS ending at position n (since they are no greater than S_n).

Searching for the maximum M_j with $S_i \geq S_j$ can be done in time $\mathcal{O}(\log n)$ when storing the values in a sorted data structure. One needs to generate at most $n-1$ links to prefixes S_j for each position $i = 1, ..., n$. Therefore the computational complexity of this algorithm for computing all the possible solutions to the LIS problem becomes $\mathcal{O}(n^2 \log n)$, or simply $\mathcal{O}(n^2)$ when the search is omitted and all positions $j < i$ are considered for each $i = 1, ..., n$. In practice there may exist few alternative solutions and using the binary search option may be more efficient. However, the output size N may be exponential, so in the worst case an algorithm that outputs all possible LIS solutions takes time $\mathcal{O}(n^2) + (\mathcal{O}(1))^n$.

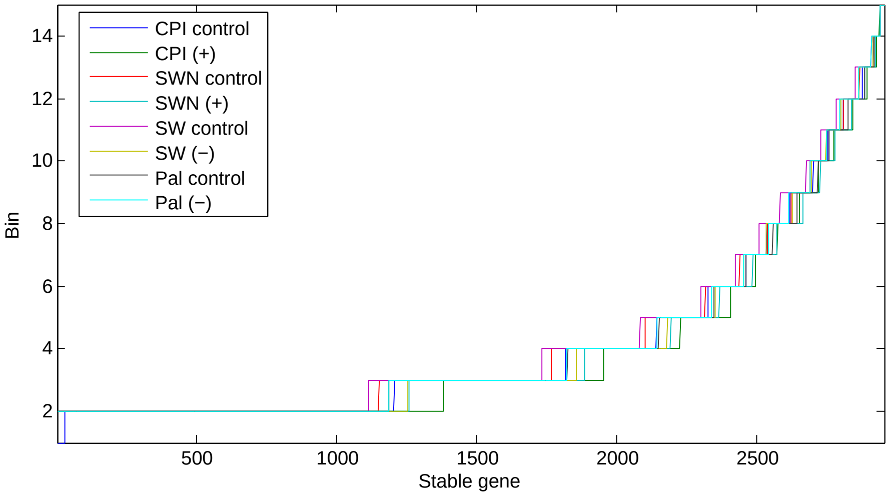

Figure 1 *Phalaris* stable genes expression level distribution. The genes are ordered by increasing expression on the x-axis, and the y-axis shows the expression bin of the x:th gene in the control/stress pair of the respective cultivar.

Stable genes on *Phalaris* data

We employed the stable genes framework to identify transcript sets whose expression remains constant (with respect to each other) between the control and stress experiments. Comparing each cultivar's stress and control experiment, we identified $7,800 - 11,400$ stable transcripts per cultivar. The intersection of those sets yields roughly $3,000$ transcripts that remain stable in cultivar during salt stress. These transcripts represent a wide range of read counts. Up to 500 of the stable transcripts have maximum norm distance 1.0 which is only about 15% of the stable transcripts, compared to about 25% transcripts with max. norm distance 1.0 in the entire data. The mode distance of the 500 stable transcripts is at most 1, thus they are a separate set from the DE candidates and represent genes whose expression remains stable during salt stress in all cultivars. The expression distribution (bins) of the stable genes are visualized in Figure 1. The stable set represents genes having a wide range of expression levels.

Additional RoDEO method comparison

For evaluating the methods, we use the MicroArray Quality Control (MAQC) III RNA-seq dataset [2] (also known as SEQC). This dataset has been previously applied in a comprehensive evaluation of existing DE methods [3]. The data consists

of two samples, one for human reference RNA and one for human brain reference RNA, with five replicates per sample. Each sample was sequenced as 100 bases paired-end reads on a HiSeq2000 instrument. The methods were run on all genes, while the DE results were evaluated only on the roughly one thousand genes with available qRT-PCR quantification, provided by the MAQC project [4].

Figure 2 shows the methods' performance on the five-replicate SEQC dataset. The results on a single-replicate case shown in Figure 3 use the first replicate only. RoDEO does especially well on cumulative true rankings (panel iii) for the top genes in Figure 2, and is on par with the best available methods in the other aspects. This is observed for a large range of thresholds for calling genes differentially expressed from the PCR validation data (panel iv). Indeed, when the threshold increases (towards calling only the most differentially expressed genes), RoDEO results improve. Only sSeq is on par with RoDEO, being slightly better on this SEQC dataset, while RoDEO was best on the MAQC PRO dataset featured in the main manuscript. baySeq and edgeR clearly fall below these two methods. Furthermore, edgeR is not applicable in the single replicate case.

References

1 Skiena, S.: The Algorithm Design Manual. Springer (2008)

2 DeLuca, D. S., Levin J. Z., Sivachenko, A., Fennell, T., Nazaire, M.-C., Williams, C., Reich, M., Winckler, W., Getz, G.: RNA-SeQC: RNA-seq metrics for quality control and process optimization. Bioinformatics 28, pp. 1530–1532 (2011)

3 Rapaport, F. Khanin R., Liang, Y., Pirun, M., Krek, A., Zumbo, P., Mason C., Socci, N., Betel, D.: Comprehensive evaluation of differential gene expression analysis methods for RNA-seq data, Genome Biology 14 (2013)

4 Canales, R.D., Luo, Y., Willey, J.C., Austermiller, B., Barbacioru, C.C., Boysen, C., Hunkapiller, K., Jensen, R.V., Knight, C.R., Lee, K.Y., Ma, Y., Maqsodi, B., Papallo, A., Peters, E.H.H., Poulter, K., Ruppel, P.L., Samaha, R.R., Shi, L., Yang, W., Zhang, L., Goodsaid, F.M.: Evaluation of DNA microarray results with quantitative gene expression platforms. Nature biotechnology 24, 1115–1122 (2006)

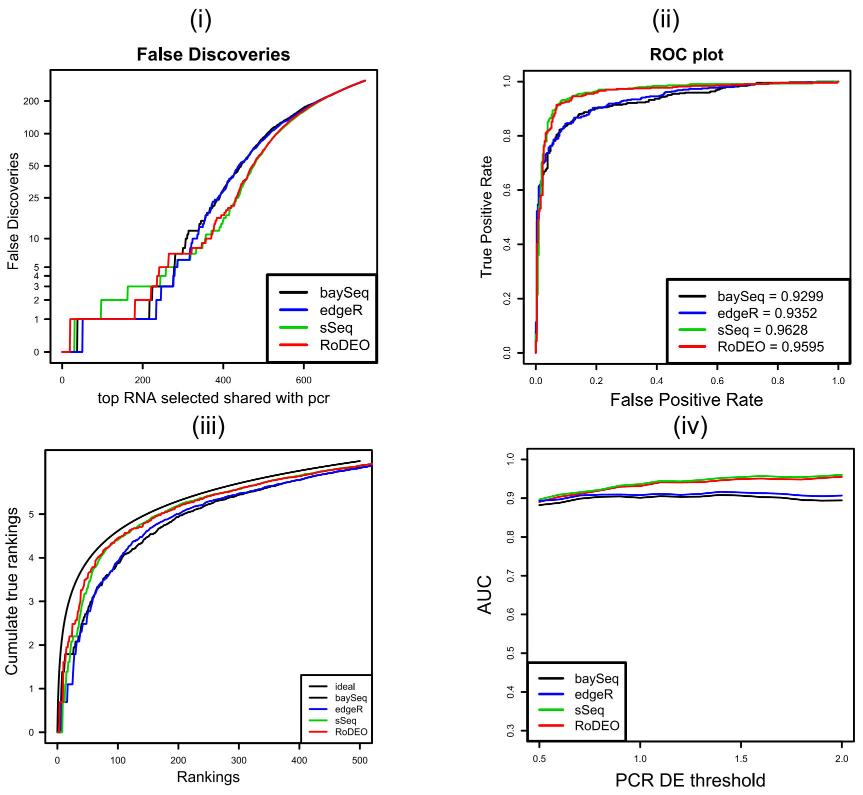

Figure 2 SEQC results on five replicates. The four panels show (i) false discoveries in the top DE genes, (ii) false positive vs. true positive rate and area under the curve (AUC) measurement, (iii) number of true DE genes with ranks $1 \ldots x$ within top x genes by the method, and (iv) AUC when varying the threshold for calling DE genes from the qRT-PCR results (threshold 1.5 is used in panels i-iii).

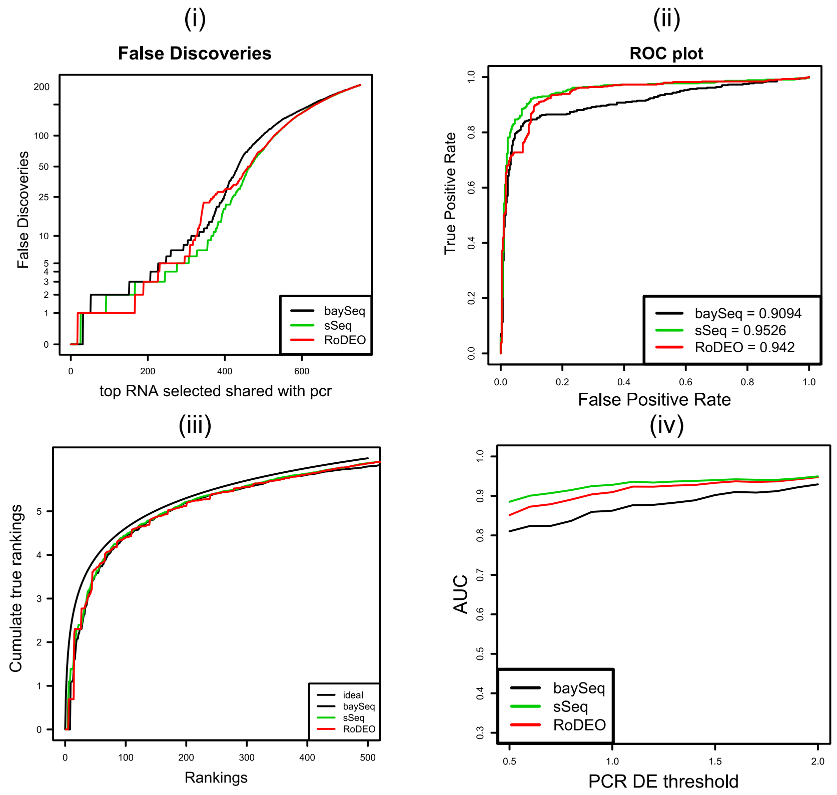

Figure 3 SEQC results on one replicate. The four panels show (i) false discoveries in the top DE genes, (ii) false positive vs. true positive rate and area under the curve (AUC) measurement, (iii) number of true DE genes with ranks $1 \ldots x$ within top x genes by the method, and (iv) AUC when varying the threshold for calling DE genes from the qRT-PCR results (threshold 1.5 is used in panels i-iii).
